# The impact of thrombosis on probabilities of death and disease progression in polycythemia vera: a multistate transition analysis of 1,545 patients

**DOI:** 10.1038/s41408-023-00960-1

**Published:** 2023-12-15

**Authors:** Tiziano Barbui, Alessandra Carobbio, Juergen Thiele, Naseema Gangat, Elisa Rumi, Alessandro Rambaldi, Alessandro M. Vannucchi, Ayalew Tefferi, IWG-MRT group, Georg Jeryczynski, Leonhard Müllauer, Rakhee Vaidya, Nanna H. Sulai, Animesh Pardanani, Dirk R. Larson, Mario Cazzola, Ilaria Casetti, Guido Finazzi, Lisa Pieri, Heinz Gisslinger, Bettina Gisslinger, Francesco Rodeghiero, Marco Ruggeri, Maria Luigia Randi, Irene Bertozzi, Francesco Passamonti

**Affiliations:** 1 FROM Research Foundation, Papa Giovanni XXIII Hospital, Bergamo, Italy; 2 Institute of Pathology, University of Cologne, Cologne, Germany; 3 Hematology Division, Mayo Clinic, Rochester, MN USA; 4 Department of Molecular Medicine, University of Pavia, Pavia, Italy; 5 Division of Hematology, Fondazione Istituto di Ricovero e Cura a Carattere Scientifico Policlinico San Matteo, Pavia, Italy; 6 Hematology and Bone Marrow Transplant Unit, ASST Papa Giovanni XXIII, Bergamo, Italy; 7 Department of Oncology and Hematology-Oncology, University of Milan, Milan, Italy; 8 Center Research and Innovation of Myeloproliferative Neoplasms (CRIMM), Department of Experimental and Clinical Medicine, Azienda Ospedaliera Universitaria Careggi, University of Florence, Florence, Italy; 9 Medical University of Vienna, Vienna, Austria; 10 S. Bortolo Hospital, Vicenza, Italy; 11 Department of Hematology, University of Padua, Padua, Italy; 12 Università degli Studi di Milano; Fondazione IRCCS Ca’ Granda Ospedale Maggiore Policlinico, Milan, Italy

**Keywords:** Myeloproliferative disease, Risk factors

## Abstract

We applied a parametric Markov five-state model, on a well-characterized international cohort of 1,545 patients with polycythemia vera (PV; median age 61 years; females 51%), in order to examine the impact of incident thrombosis on the trajectory of death or disease progression. At a median follow-up of 6.9 years, 347 (23%) deaths, 50 (3%) blast phase (BP), and 138 (9%) fibrotic (post-PV MF) transformations were recorded. Incident thrombosis occurred at a rate of 2.62% pt/yr (arterial 1.59% and venous 1.05%). The probability of death, in the first 10 years, for 280 (18%) patients who developed thrombosis during follow-up was 40%, which was two-fold higher than that seen in the absence of thrombosis or any other transition state (20%; *p* < 0.01); the adverse impact from thrombosis was more apparent for arterial (HR 1.74; *p* < 0.01) vs venous thrombosis (p=NS) and was independent of other fixed (i.e., age, prior venous thrombosis, leukocytosis) or time-dependent (i.e., progression to BP or MF) risk variables. The transition probability to post-PV MF increased over time, in a linear fashion, with a rate of 5% capped at 5 and 10 years, in patients with or without incident thrombosis, respectively. The impact of thrombosis on transition probability to death or post-PV MF tapered off beyond 10 years and appeared to reverse direction of impact on MF evolution at the 12-year time point. These observations suggest thrombosis in PV to be a marker of aggressive disease biology or a disease-associated inflammatory state that is consequential to both thrombosis and disease progression.

## Introduction

Patients with myeloproliferative neoplasms (MPNs) face diverse critical outcomes, including disease progression to myelofibrosis (MF) or blast phase disease (MPN-BP), thrombotic complications, and death [1–4]. Previous studies analyzing the risk of these events have relied primarily on conventional statistical methods, such as Kaplan-Meier survival curves or simple Cox proportional hazards regression, which examine the effect of one or more fixed variables on the time it takes for a particular event to occur. However, these techniques may not fully capture the complexities of multiple potential outcomes and time-varying covariates. In many cases, they provide survival or hazard estimates that did not consider the intermediate disease states and transitions occurring before the final adsorbing state. As a result, novel approaches are needed to understand and predict outcomes in MPN patients more comprehensively. Multistate models offer numerous advantages over conventional statistical analysis when studying life history processes, making them particularly valuable in investigating MPN diseases like polycythemia vera (PV), essential thrombocythemia (ET), or primary myelofibrosis (PMF). These models are well-suited to address critical disease endpoints, as they can handle competing risks and account for the probability of multiple outcomes occurring simultaneously.

In the current study, we present data generated by multistate models from a large series of 1,545 patients with PV and obtained from the International Working Group for Myeloproliferative Neoplasms Research and Treatment (IWG-MRT). Prior analyses on this patient cohort, was conducted by Tefferi et al. [5], focusing on survival and occurrence of acute myeloid leukemia. However, this analyses only considered overall survival and MPN-BP transformation as a competing risk, neglecting potential interactions between different states and transitions among them. We address here this limitation by estimating the transitions between states and examining the role of incident arterial and venous thrombosis in modulating the occurrence of post-PV MF, MPN-BP, and death. By means of a time-dependent approach, we investigated the factors independently associated with the evolution of post-PV MF, MPN-BP, and death, considering intermediate states recorded during follow-up.

## Patients and methods

### Patients

The present study is a re-analysis of data from the aforementioned IWG-MRT cohort of 1,545 patients with strictly World Health Organization-defined PV [1]. Data at the time of PV diagnosis as well as major outcomes occurring during the follow-up were recorded. Only objectively proven major arterial and venous events were considered. Diagnosis of progression to post-PV MF was established by the corresponding clinical features. The latter included worsening of anemia, increase in splenomegaly, and/or overt leukoerythroblastosis consistent with advanced MF with myeloid metaplasia [2]. Transformation to MPN-BP met criteria ( ≥ 20% blasts) in agreement with the International Consensus (ICC) [3, 4] and WHO classification systems.

### Statistical methods

**Fig. 1:**
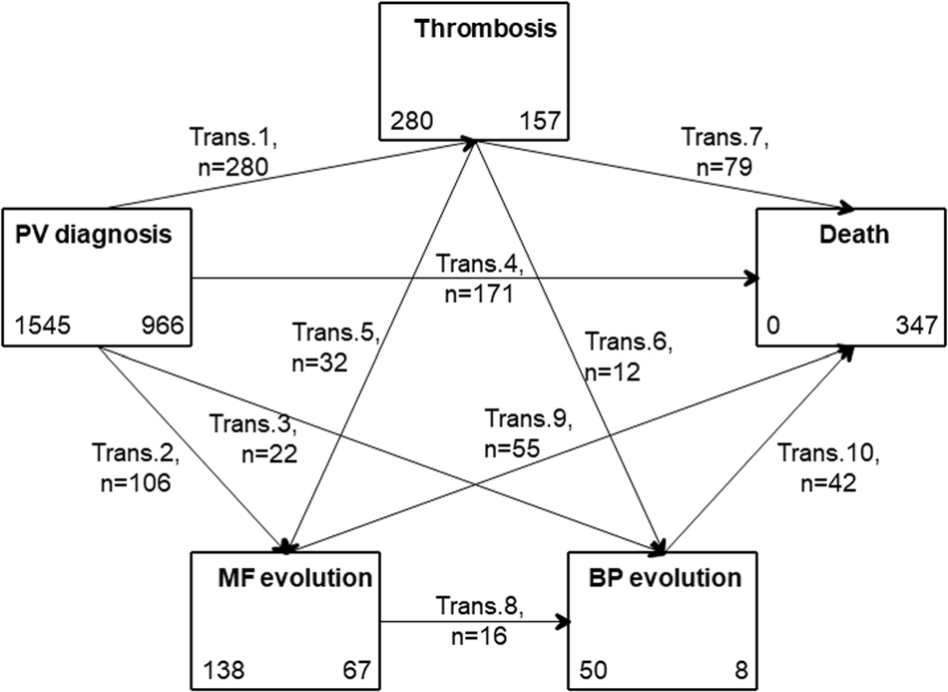
Outline of the 5-states model for the clinical course of PV patients. The five states, graphed by boxes, are (1) diagnosis of PV, (2) thrombosis, (3) MF (4) BP and (5) death. In each box is quoted the number of patients starting (left) and ending (right) in the state. The following 10 transitions (graphed by arrows) are possible: from PV to thrombosis (trans.1), to MF (trans. 2), to BP (trans. 3), to death (trans. 7); from thrombosis to MF evolution (trans. 5), to BP (trans. 6), to death (trans. 7); from MF to BP (trans 8) or death (trans 9); from BP to death (trans. 10). On each arrow is quoted the number of patients involved in the corresponding transition.

A five-state parametric Markov survival model was used to describe the clinical course of PV, following the approach proposed by Crowther et al. [6] (Fig. 1), using the “multistate” package in STATA software. States included ranged from diagnosis to death with in-between events that could be thrombosis, progression to post-PV MF or MPN-BP. Depending on the path taken to reach the state of interest, transitions from one state to another could be direct or indirect and related probabilities have been compared. In order to identify risk factors for mortality and disease progression, Cox regression models were performed adjusting either for fixed effects (risk factors known at PV diagnosis) and for variable (time-dependent) effects, such as the occurrence of intermediate states.

## Results

### Presenting features

The characteristics of the cohort recruited (Table 1-S) reflect well the heterogeneity of epidemiologic and clinical features that are found in the routine clinical practice of PV. Arterial and venous thrombosis before or at diagnosis was documented in 246 (16%) and 114 (7.4%) patients, respectively. Among cardiovascular risk factors, arterial hypertension was the most prevalent (*n* = 683, 46%). Treatment was according to individual physician discretion and cytoreductive drug or aspirin use was documented in 1129 (73%) and 1281 (84%) patients, respectively.

#### Thrombotic events after diagnosis

Median follow-up was 6.9 years. Post-diagnosis total thrombosis rate was 2.62% pt/yr. Arterial or venous thrombosis occurred in 184 (12%, rate:1.59% pts/yr) and 137 (9%, rate:1.05% pts/yr) patients, respectively (Table 2-S). In addition, prior arterial events were associated in 75% of cases with subsequent arterial events and in the remaining 25%, with venous thrombosis including splanchnic vein thrombosis. Moreover, patients with prior venous events had recurrences in venous and arterial site in 61% and 39% of cases, respectively.

### Five-state model and transitions

Half of the deaths (49%) were recorded directly from the diagnosis of PV without any intermediate transition, while 23% and 28% were mediated by the transition to thrombosis or evolution to post-PV MF/MPN-BP, respectively (Fig. 1). The evolution to post-PV MF occurred directly from the diagnosis in 85% of cases or via thrombosis in the remaining 15%. The subsequent status from post-PV MF was MPN-BP or death and pertained to half of cases. Transition from diagnosis to MPN-BP occurred directly in 44% of cases and through the movement to post-PV MF in 32% of cases. Two-hundred-eighty patients developed at least one major thrombotic event directly after diagnosis: venous thrombosis in 132 and arterial thrombosis in 178. Of note, the number of thrombotic events included in the 5-state model was 280, whereas the original analysis by Tefferi et al. [5] included 290 vascular episodes. This discrepancy was due to the Markov assumption used (i.e., the clock-forward approach), which did not allow for backward transitions; thus, 10 thrombotic events were not counted because they occurred after the transition to post-PV MF. From thrombosis status, the frequency to transition to death, post-PV MF and MPN-BP was ascertained in 79 (28%), 32 (11%), and 12 (4%) patients, respectively.

### State occupation probabilities

**Fig. 2:**
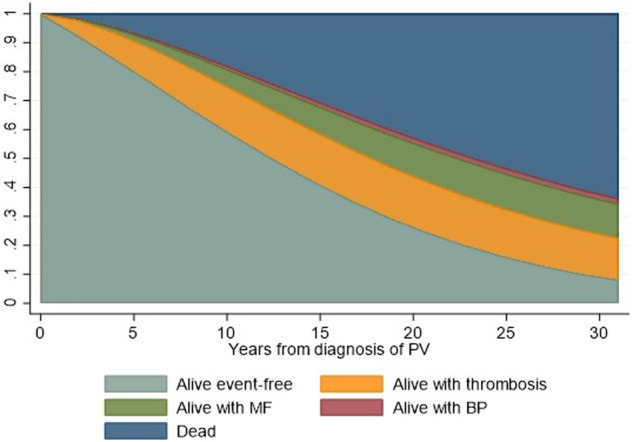
State occupation probabilities. Stacked plot of state occupation probabilities of being alive free-from thrombosis, evolution into overt MF, BP, or having died as a function of time since diagnosis of PV. The figure shows the state occupation probabilities, which are the probability of being in a state at a certain time. The stacked presentation allows to compare the four different probabilities simultaneously. At the time 0 (diagnosis), the probability of being alive and free-from events is 100%. During the course of the diseases, this probability gradually decreases in favor of other states occupation.

The probabilities of being event-free during follow-up or having thrombosis, post-PV MF, MPN-BP, or death are illustrated in Fig. 2. The probability of being event-free 10 years after diagnosis was 60% and decreased to 10% after 30 years of observation; the overall mortality was over 40%. While the probability of developing a thrombosis tended to increase during the first 5 years, it remained stable at around 15–20% thereafter and until the end of follow-up. As expected, the likelihood of developing post-PV MF was evident after the first 5 years and then increased to a stable 10–15%.

### Thrombosis state and the probability of disease progression and death

**Fig. 3:**
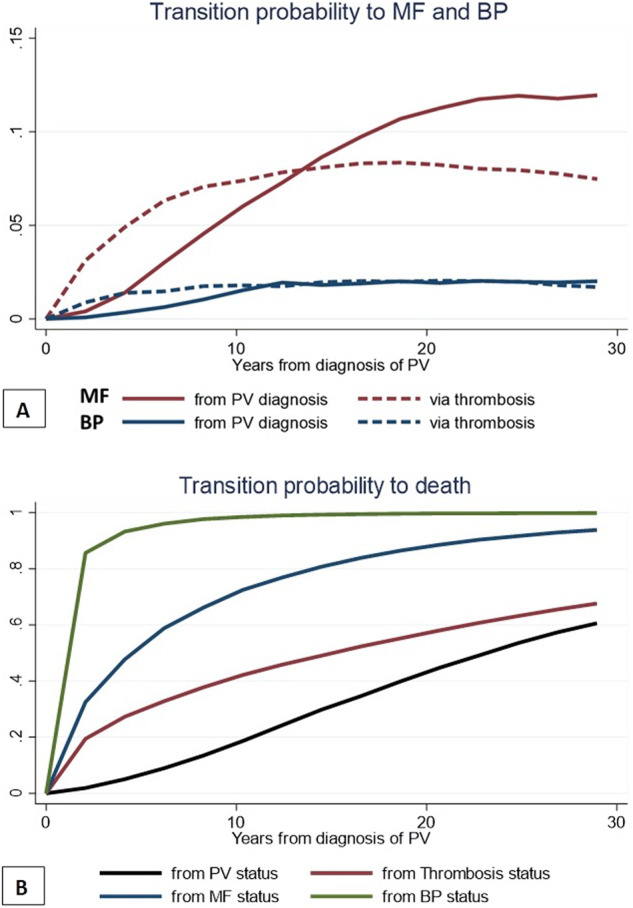
Direct and mediated probabilities to evolution in MF and BP (A) and death (B). Transition probabilities are defined as the probability of going from a given state to the next state in a Markov process. Panel **A** reports the direct (i.e., from PV diagnosis) and after thrombosis probability to evolve into overt MF or BP. In panel **B** are illustrated the direct and indirect (via thrombosis, evolution in MF or BP) transition probabilities to death (absorbing state) over time.

Three hundred and forty-seven (23%) deaths, 50 (3%) transitions into MPN-BP, and 138 (9%) progressions to post-PV MF) were recorded during the follow up. The transition probability to post-PV MF (red lines) directly from PV diagnosis showed a progressive and linear increase over time. After 5 years, post-PV MF occurred in 5% of patients with thrombosis while this probability was reached later, after 10 years, in patients who directly experienced MF without thrombosis. An earlier acceleration from thrombosis to MPN-BP (blue curves) was also observed compared to the direct transition from PV diagnosis (2% vs. 0.5% at 5 years). However, the accelerating effect of thrombosis on disease progression tapered off after 10 years, as shown by the overlap of the two curves. (Fig. 3a)

The expected probability of death of patients without passing through intermediate states (black curve) or after passing through thrombosis, post-PV MF or MPN-BP is shown in Fig. 3b. The probability of death in the first 10 years for the 280 (18%) patients who developed thrombosis during follow-up was twice (40%) that of direct mortality in the absence of thrombosis or any other transition state (20%; *p* < 0.01).

### The transition state of thrombosis was independently associated with increased mortality

Risk factors for the probability of progression to post-PV MF, MPN-BP, and death were explored considering both fixed variables at diagnosis and events occurring over time during follow-up (Table 1). The results showed a statistically significant effect of age in predicting mortality, with patients older than 65 years having a higher risk of progression to MPN-BP (*p* = 0.031). A history of venous thrombosis was also independently associated with death (*p* = 0.008). However, no significant effect of prior venous or arterial thrombosis on the risk of post-PV MF or MPN-BP was observed.

**Table 1:** Fixed and time-dependent risk factors for MF, BP evolutions, and mortality.

	MF evolution (*n* = 138)				BP evolution (*n* = 50)				Death (*n* = 347)			
Covariate	HR	*p*	95% CI		HR	*p*	95% CI		HR	*p*	95% CI	
*Fixed variables at diagnosis*												
Male sex	1.08	0.749	0.68	1.72	1.07	0.816	0.61	1.88	1.08	0.596	0.81	1.43
Age<40 y	1 (ref)				1 (ref)				1 (ref)			
40-64 y	2.14	0.086	0.90	5.10	1.45	0.502	0.49	4.26	**2.31**	**0.025**	**1.11**	**4.81**
>=65 y	2.10	0.145	0.77	5.67	**3.28**	**0.031**	**1.12**	**9.60**	**11.60**	**0.000**	**5.52**	**24.41**
Previous arterial thrombosis	1.50	0.204	0.80	2.81	0.93	0.870	0.39	2.21	1.35	0.090	0.95	1.91
Previous venous thrombosis	0.74	0.580	0.26	2.13	0.72	0.655	0.17	3.03	**1.86**	**0.008**	**1.18**	**2.93**
CV risk factors					—							
Smoke	0.49	0.098	0.21	1.14	—				1.36	0.100	0.94	1.95
Diabetes	1.73	0.154	0.81	3.70	—				0.93	0.779	0.58	1.50
Hyperlipidemia	0.69	0.323	0.33	1.45	—				1.19	0.344	0.83	1.71
Hypertyension	1.03	0.911	0.65	1.62	—				1.23	0.184	0.91	1.66
*Time-dependent variables in the f-up*												
Arterial thrombosis	1.01	0.979	0.49	2.06	0.99	0.985	0.40	2.46	**1.74**	**0.009**	**1.15**	**2.65**
Venous thrombosis	1.16	0.719	0.51	2.62	**2.09**	**0.086**	**0.90**	**4.85**	1.32	0.255	0.82	2.14
MF evolution	—				**9.06**	**0.000**	**4.36**	**18.86**	**4.83**	**0.000**	**3.23**	**7.22**
BP evolution	—				—				**16.63**	**0.000**	**7.37**	**37.54**

Statistically significant (i.e. *p* < 0.05) covariates are shown in bold.

A time-dependent analysis approach was used to estimate the risk of variables occurring in the follow-up. A trend to develop MPN-BP was associated with venous thrombosis (*p* = 0.086), whereas no significant effects were seen for either arterial or venous thrombosis, on the risk of progression into post-PV MF. Interestingly, incident arterial thrombosis emerged as a significant risk factor for death (*p* = 0.009), with a hazard ratio (HR) of 1.74, independently from the risk associated with post-PV MF (HR 4.83) or MPN-BP (HR 16.63).

## Discussion

The current study is based on a very large and well-characterized series of 1545 patients with WHO-defined PV and constitutes re-analyses by a multistate approach that revealed an impact from incident thrombosis on probabilities of death and disease progression into post-PV MF and MPN-BP. The study cohort was representative of routine clinical practice in PV and had clinical and epidemiological characteristics ensuring the generalizability of the present results.

During the median follow-up of 6.9 years, the probability of being event-free (i.e., without experiencing thrombosis or evolution into post-PV MF, MPN-BP, or death) at 10 years after diagnosis was 60%.

The overall thrombosis rate after the initial diagnosis of PV was 2.62% per patient-year, with arterial and venous thrombosis rates of 1.59% and 1.05% per patient-year, respectively. This probability tended to increase during the initial 5 years after diagnosis and stabilized at around 15–20% thereafter. This observation, obtained by multistate models which simultaneously evaluated other intermediate events as competing risks, is in line with findings of prior observational and registry studies [7–9] confirming that the risk of thrombosis is higher shortly after the diagnosis of MPNs, and stabilizes over time in the absence of other significant factors.

Vascular events were associated with an accelerating effect on the onset of post-PV MF and MPN-BP in the first 10 years after PV diagnosis, and this effect diminished thereafter. This was clearly demonstrated by the overlap of the two cumulative event curves, suggesting that other factors became more influential in driving disease progression in the later stages of PV [10, 11]. The probability of progression to post-PV MF directly from PV diagnosis showed a progressive and linear increase from 1% to 5% at 5 and 10 years, respectively. This risk increased to 5% earlier at 1 year in patients with incident thrombosis. Similarly, the occurrence of MPN-BP was accelerated in the first years if it occurred via thrombosis reaching 2% compared to the direct transition from PV diagnosis (0.5%). Interestingly, patients with venous thrombosis were more likely to develop MPN-BP.

Risk factors of disease progression and mortality were assessed both at diagnosis and during the follow-up through a time-dependent analysis. Age was a statistically significant predictor of mortality, with patients over 65 years having a higher risk of progressing to MPN-BP. A history of venous thrombosis was found to be independently associated with increased risk of death, whereas no significant effect was noted for history of prior venous or arterial events on the risk of post-PV MF or MPN-BP. Utilizing time-dependent analysis approach, the current study identified incident arterial thrombosis as a risk factor for death, independently of the more potent risk factors associated with post-PV MF and MPN-BP. These observations support the notion that clonal hematopoiesis, involving mutations in both driver and non-driver genes, induces a long-lasting inflammatory state that not only increases the risk of thrombosis, but also may promote stem cell expansion, thereby driving disease progression from a chronic disease state to more advanced stages [12–15].

Whether these results can also be reproduced in patients diagnosed with the current 2016 WHO criteria, which encompass cases with masked PV, remains to be explored in appropriately sized cohorts with a prolonged follow-up period.

Taken together, the current multistate analysis of PV patients who have experienced multiples states of transition, has provided new information on the role of incident thrombosis in influencing mortality and transformation to post-PV MF and MPN-BP. Our data represents integration of results from Cox regression analysis, which are primarily designed for analyzing time-to-event data with a single event of interest and does not directly handle multiple competing risks. The primary limitation of this retrospective study is the potential for case selection bias and incomplete data. We couldn’t accurately assess the impact of cytoreductive drugs on post-diagnosis events due to missing critical information. Additionally, our results were obtained from the same PV database used in Tefferi et al. survival and blast phase analysis [5], raising the possibility of “self-confirmation bias.”

In conclusion, this study, based on a large and well-characterized PV patient cohort, offers new insights into how arterial and venous thrombosis affect mortality. It may also generate hypotheses regarding myelofibrosis, blast phase development, and mortality, which could inform clinical practice.

### Supplementary information

Supplementary material

## Data Availability

Data is available on request due to privacy/ethical restrictions.
