# Supplementary material for: The impact of thrombosis on probabilities of death and disease progression in polycythemia vera: a multistate transition analysis of 1,545 patients
Source: Blood Cancer J. 2023 Dec 15;13(1):187. doi: 10.1038/s41408-023-00960-1 (PMC10724164; doi:10.1038/s41408-023-00960-1)
Supplement: Supplementary file 1 — Supplementary material [file 41408_2023_960_MOESM1_ESM.docx]

**SUPPLEMENTARY MATERIAL**

**Table 1-S. Patients’ characteristics at PV diagnosis**

| **Characteristic** | ***N*** | **Value** |
| --- | --- | --- |
| **Age, years, median (range)** | 1545 | 61 (18-95) |
| *< 40 years, n (%)* | 1545 |  |
| *40-64 years, n (%)* | 1545 |  |
| *≥ 65 years, n (%)* | 1545 |  |
| **Sex M/F, n (%)** | 1545 | 760/785 (49/51) |
| **Hemoglobin, g/dL, median (range)** | 1545 | 18.4 (15.1-26.5) |
| **Hematocrit, %, median (range)** | 1545 | 55 (36-78) |
| **Leukocyte count, x 10^9^/L, median (range)** | 1545 | 10.4 (3-171.6) |
| **Platelet count, x 10^9^/L, median (range)** | 1545 | 466 (7-2370) |
| **JAK2*V617F* mutation, n (%)** | 1268 | 1239 (98) |
| **History of tobacco use, n (%)** | 1301 | 206 (16) |
| **History of diabetes, n (%)** | 1149 | 97 (8) |
| **History of hyperlipidemia, n (%)** | 1073 | 196 (18) |
| **History of hypertension, n (%)** | 1388 | 638 (46) |
| **Arterial thrombosis before/at diagnosis, n (%)** | 1545 | 246 (16) |
| *AMI, n (%)* | *1545* | *65 (4)* |
| *Stroke/TIA, n (%)* | *1545* | *138 (9)* |
| *PAT, n (%)* | *1545* | *29 (2)* |
| *Other/Unknown, n (%)* | *1545* | *14 (1)* |
| **Venous thrombosis before/at diagnosis, n (%)** | 1545 | 114 (7) |
| *DVT/PE, n (%)* | *1545* | *78 (5)* |
| *SPLANCHNIC, n (%)* | *1545* | *30 (2)* |
| *Other/Unknown, n (%)* | *1545* | *6 (0)* |

**Table 2-S. Major events in PV patients**

| **Major events in the follow-up** | ***N evaluable*** |  |
| --- | --- | --- |
| **Years of follow-up, median (range)** | 1545 | 6.9 (0-39) |
| **Total thrombosis, n (%)**  **Incidence rate, % pat/year (95% CI)** | 1545 | 290 (19)  2.62 (2.34-2.94) |
| **Arterial Thrombosis, n (%)**  **Incidence rate, % pat/year (95% CI)** | 1545 | 184 (12)  1.59 (1.38-1.84) |
| *AMI, n (%)* | *1545* | *35 (2)* |
| *Stroke/TIA, n (%)* | *1545* | *104 (7)* |
| *PAT, n (%)* | *1545* | *34 (2)* |
| *Other/Unknown, n (%)* | *1545* | *11 (1)* |
| **Venous Thrombosis, n (%)**  **Incidence rate, % pat/year (95% CI)** | 1545 | 137 (9)  1.05 (0.88-1.25) |
| *DVT/PE, n (%)* | *1545* | *88 (6)* |
| *SPLANCHNIC, n (%)* | *1545* | *27 (2)* |
| *Other/Unknown, n (%)* | *1545* | *22 (1)* |
| **MF evolution, n (%)**  **Incidence rate, % pat/year (95% CI)** |  | 138 (9)  1.13 (0.95-1.33) |
| **BP evolution, n (%)**  **Incidence rate, % pat/year (95% CI)** | 1545 | 50 (3)  0.40 (0.30-0.52) |
| **Death, n (%)**  **Incidence rate, % pat/year (95% CI)** | 1545 | 347 (23)  2.74 (2.46-3.04) |
